# Exploring Staff Perceptions and Experiences in Services Providing Different Levels of Active Support

**DOI:** 10.1111/jir.70047

**Published:** 2025-09-23

**Authors:** Jill Bradshaw, Beckie Whelton, Julie Beadle‐Brown, Lisa Richardson, Jennifer Leigh

**Affiliations:** ^1^ Intellectual Disabilities Research Institute (IDRIS) University of Birmingham Birmingham UK; ^2^ Tizard Centre, School of Psychology University of Kent Canterbury UK; ^3^ California Community Living Network San Francisco California USA; ^4^ School of Policy, Sociology and Social Research University of Kent Canterbury UK

## Abstract

**Introduction:**

Outcomes for people with intellectual and developmental disabilities, particularly those with more severe and complex needs, depend on the quality of support they receive from staff. This paper explores staff perspectives on skilled support and relationships with training and experience.

**Methods:**

Questionnaires were received from 93 staff working in 28 supported accommodation services in which observations of the quality of support had also been conducted. Staff were asked about experience, training and views on skilled support. Content analysis was used to code written responses. Statistical analysis explored relationships between staff responses, and the quality of support was observed.

**Results:**

Staff perceived improving the quality of life of people they supported as key. All staff considered themselves as being at least partially skilled, with the majority associating being skilled with length of experience and attendance at training. Specific training was rarely mentioned, and receipt of training was not associated with the provision of better quality of support. Practice leadership was rarely mentioned.

**Conclusions:**

In most cases, staff showed awareness of the principles set out in policy, but their reflections did not match observed practice.

## 1 Introduction

People with intellectual and developmental disabilities in adult social care settings often experience poor quality of life outcomes (Esteban et al. 2021; Beadle‐Brown et al. 2015). Staff play a paramount role in ensuring that people experience good outcomes (Landesman‐Dwyer et al. 1980; Flynn et al. 2018); however, relatively little is known about staff views around quality support and their perceptions of what might be important in order to achieve good outcomes for people with intellectual and developmental disabilities.

Topping et al. (2022, 4259) identified five key factors that were considered by staff themselves to influence the quality of support—‘being the right person for the role, delivering quality support in practice, working well together, maintaining and improving quality support and considering the broader context’. Their work also highlighted the importance of the support worker ‘recognising the person as an individual and respecting their autonomy’.

Factors related to teamwork and staff support have also been highlighted as important for effective staff teams, for example, shared focus on client outcomes, good communication and effective leadership (Gomes and McVilly 2019).

Empirical research has identified that if staff work in a facilitative way, enabling and empowering individuals using an approach called ‘active support’ (doing *with* not just *for* or *to*), then outcomes are better (Beadle‐Brown et al. 2020; Beadle‐Brown et al. 2015; Flynn et al. 2018; van Herwaarden et al. 2025). However, research has found that, on average, only between one third and one fifth of people receiving support in social care settings receive consistently good support (Netten et al. 2010; Beadle‐Brown et al. 2015; Bigby et al. 2017).

This is particularly true for those with more severe disabilities, who are often completely reliant on others to have, and to take advantage of, opportunities at home and in the community (Beadle‐Brown et al. 2015). For those in this group, staff need a variety of different skills to meet the needs of individuals and to support good quality of life outcomes; for example, communication skills and skills in managing complex health conditions.

Despite the critical and complex roles that staff play in the quality and outcomes of services, research is sparse. Previous research found that qualifications did not generally equate to better support for people with intellectual disabilities, although having had training in the approach referred to as Active Support (Mansell and Beadle‐Brown 2012) was associated with better outcomes (Flynn et al. 2018; Bigby, Bould, and Beadle‐Brown 2019; Bigby, Bould, Iacono, and Beadle‐Brown 2019a, 2019b; Bould et al. 2019). If staff received practice leadership from front‐line managers and worked in an organisation where senior managers understood and supported the implementation of practices such as active support, then the quality of support and outcomes was greater (Beadle‐Brown et al. 2015; Bigby, Bould, and Beadle‐Brown 2019; Bigby, Bould, Iacono, and Beadle‐Brown 2019a, 2019b; Bould et al. 2019).

Relatively little is known about how staff understand what services are trying to achieve, how they perceive their roles and the support needed by people with intellectual disabilities and whether this has an impact on their practice. Previous research has shown that staff typically prioritise direct care (McConkey and Collins 2010), may have more focus on what they are *not* allowed to do (Perry et al. 2003) and often use trial and error (Bradshaw and Goldbart 2013; Windley and Chapman 2010). More recent research has often focused on staff providing services to people who present with behaviours that challenge and has found that staff with higher levels of self‐reflection and higher educational levels were related to better quality of life for the people they supported (Bruinsma et al. 2022).

As part of a larger study on the quality and outcomes of skilled support, a questionnaire was conducted with staff in small group homes and supported living settings (supporting from one to six people) in different geographical areas in England. More information about the wider project can be found in (Beadle‐Brown et al. 2016).

The aim of this paper is to explore staff experiences, training, views of skilled support and the relationships between these and the quality of support received. Specifically, this paper seeks to answer the following research questions:

1. How do staff perceive the aims of the service in which they work?
2. How do staff describe their role and the support they provide to people?
3. How do staff describe the type of skilled support needed by the people they support?
4. What do staff report as important in helping them to become skilled?
5. Do staff perceptions, descriptions and reports differ when they are working in a service that provides good active support?

## 2 Methodology

The data presented in this paper were collected as part of a larger study looking at the nature, costs and outcomes of skilled support for people with severe intellectual disabilities and complex needs (Beadle‐Brown et al. 2016, 2020). The study was a one point in time, mixed methods study.

### 2.1 Participants

All staff (*N* = 372) from 28 services across 16 organisations providing support for people with severe or profound intellectual disability and were either autistic had multiple physical disabilities or showed behaviour described as challenging were invited to participate in the survey. Surveys were returned from 93 staff members (25% return rate). Characteristics of the 85 people supported by these services can be seen in Table 1.

**TABLE 1:** Characteristics of the people supported (*n* = 85).

Characteristic	Mean (range)
Age—mean (range and SD)	Mean 46 years (20–82)
Mean Adaptive Behaviour Scale	114 (27–248)
Mean Aberrant Behaviour Checklist	39 (0–133)

Characteristic	%
Gender—male	55%
Ethnicity—White British	84%
Physical disability	54%
Autism	41%
Epilepsy	34%
Social impairment	76%

There were no significant differences in the characteristics of people supported between services from which staff returned questionnaires and those that did not. Further details of the wider sample can be found in (Beadle‐Brown et al. 2016).

Thirty‐two percent (*n* = 9) of the services from which staff returned questionnaires were registered residential services, and 68% (*n* = 19) were supported living services. Seventy‐one percent (*n* = 20) of services were nominated by their organisation as providing skilled support. The remaining services had been randomly selected from the Care Quality Commission list of registered services and had agreed to participate in the study. The sample was selected to include a good geographical spread of services in England. Thirty‐six percent (*n* = 10) were located in the Northeast, 25% (*n* = 7) in the South West, 21% (*n* = 6) in the South East and 18% (*n* = 8) were in London.

### 2.2 Data Collection, Measures and Procedures

#### 2.2.1 Staff Characteristics, Experiences and Views

Staff characteristics (e.g., age group, length of service and previous training) were collected using Part 1 of the Staff Experiences and Satisfaction Questionnaire (Beadle‐Brown et al. 2003; Mansell et al. 2008). Part 1 contains only descriptive and qualitative data. Additional open response questions explored staff views of the aims of the service, the type of skilled support they thought the people they supported needed and what they needed as staff to be able to provide this support. These three questions (based on the research questions for the wider study and reviewed by the project advisory group) allowed us to indirectly explore staff members' perceptions of their own roles, the language they used to talk about the people they supported and to some extent their attitudes and whether they showed awareness of concepts such as active support and practice leadership.

All members of staff were invited (via their managers) to complete the questionnaires and either return these in a prepaid envelope or leave them for collection by researchers. Completion of staff questionnaires was voluntary.

#### 2.2.2 Quality of Support

As described in (Beadle‐Brown et al. 2016), the quality of support was rated using the Active Support Measure (ASM) (Mansell et al. 2005). Structured observations took place in each service between 4 and 6 PM, at the end of which time the ASM was completed for each person with a learning disability who was present at the time and for whom consent or consultee advice had been gained. This 15‐item scale focuses on the opportunities for involvement and the skills with which staff provided and supported these opportunities. Each item is scored on a scale of 0 (*poor, inconsistent support/performance*) to 3 (*good consistent performance*) with a maximum score of 45. For each person, a percentage of the maximum score was calculated and an ASM category derived—weak (ASM score < 33.33%), mixed (33.3366.66%) and good (66.67% and above). Internal consistency of the total scale was over 0.90, and there was a strong positive correlation on total score between observers (Spearman's rho = 0.848) and satisfactory Kappa values for the categorisation into ASM group (average Kappa—0.668). For this paper, we have used an average percentage score that was calculated across all people supported in each setting and then categorised as either good (ASM of > 66.66) or weak/mixed (ASM score of ≤ 66.66) active support.

#### 2.2.3 Analysis

Content analysis (Drisko and Maschi 2015) was used to code the qualitative free text responses. Content analysis involves classifying text into categories so that text with similar meanings is grouped together and can be efficiently described and analysed (Weber 1990). In this conventional content analysis (Hsieh and Shannon 2005), stages included reading all the responses, highlighting those which captured key concepts, combining these into codes that reflected more than one key thought and then organising and grouping codes into meaningful clusters. All data were jointly coded by the first and third authors, who together made the decision around whether or not data was codable. Very few answers could not be coded. Answers could not be coded if they were too general in relation to the question being asked. Using SPSS Version 29, descriptive statistics were used to explore staff characteristics and the frequencies of reported views. Using a similar approach to that used by Dodevska and Vassos (2013) and Bigby, Bould, and Beadle‐Brown (2019), Chi‐square or Mann–Whitney *U* tests were used to explore differences and associations, with Bonferroni corrections applied when multiple comparisons were needed.

#### 2.2.4 Ethical Approval

Ethical approval was gained from the Social Care Research Ethics Committee. Local Authority research governance approval was gained initially from University of Kent, and evidence of approval was sent to all local authorities where services were likely to be included.

## 3 Results

### 3.1 Characteristics of Staff

Table 2 provides information about the staff who completed the questionnaire.

**TABLE 2:** Characteristics of staff (*N* = 93).

Characteristic	%
Gender—female	72%
Ethnicity—White British	68%
Age—40+ years	53%
Previous experience—worked in ID/MH	35%
Experience in ID—more than 5 years' experience	58%
Role—support worker or senior support worker (including bank staff)	92%
Role—deputy/team leader	7%
Length in current service—more than 5 years' experience in current service	27%

Ninety‐three questionnaires were returned from staff across 28 services, with between one and seven questionnaires collected from each service. Forty‐eight percent of staff who returned the questionnaire worked in services rated as providing consistently good active support. All the questions were optional, which is why participant response numbers do not always equal the total number of participants. There were no significant differences at *p* < 0.05 in terms of staff characteristics between services rated as showing good active support (*n* = 16) and weak or mixed active support (*n* = 12).

### 3.2 How Do Staff Perceive the Aims of the Service in Which They Work and Do Perceptions Differ When They are Working in a Service Providing Good Active Support?

Most participant responses to the question about the aims of the service could be coded in terms of different categories of aims. Only three of 89 responses were so general that they could not be coded in any more detail (e.g., stating that the aims of the service were ‘to support people with learning disabilities’). In many of the responses, staff appeared to focus on their role as staff—what they did—rather than the aim of the service, although of course these are not unconnected.

Of the 89 staff who answered the question, 88% (*n* = 79) included reference to the individuals supported in their responses. Eight percent provided a response that was coded as service focused (e.g., ‘To give support in all areas in which the service requires’), 6% referenced an organisational focus (e.g., ‘to meet the organisation's aims and objectives’). Nine percent of staff noted that the aim of the service was to provide high quality services (e.g., ‘to give right quality support’ and ‘to give the best quality care’). Eighty‐two percent (*n* = 73) of staff mentioned quality of life or a specific element of quality of life in their response; 28% focused on only one element of quality of life. Nineteen percent talked about quality of life in general terms and did not go into detail about specific elements of quality of life (e.g., ‘to live a full life’) (see Table 3).

**TABLE 3:** Examples of themes around the service aims and the role of staff (*N* = 89).

Theme	%	Quote
Mentions QOL *n* = 73	82%	
Refers to quality of life in general terms	19%	‘To support service users to live a full life’
Referred to one specific QOL domain	28%	
Referred to two or more QOL domains	45%	
Physical well‐being (e.g., needs met, being healthy, safety and access to healthcare)	20%	‘To ensure the health and wellbeing of the people I work with’ ‘To ensure health and safety of all the people I work with’
Emotional well‐being (e.g., happiness, feeling calm and fulfilment)	16%	‘The best I can do to make them happy’ ‘To make sure our residents achieve confidence in the society’
Material well‐being (e.g., getting a job)	2%	‘To get a paid job’
Self‐determination (independence/independent living, choice and control)	58%	‘To live an independent life as much as possible’ ‘Allow them to choose who they live with and to be around people they choose’ ‘To provide choice, independence’
Social Inclusion (e.g., accessing the community—activities, facilities, services—and participating in community events and activities)	20%	‘Social networking’ ‘Be a part of their community’ ‘Access the community and use the facilities’
Interpersonal relationships (e.g., family and friends)	3%	‘Marry’
Personal development (e.g., learning new skills and becoming more independent)	22%	‘Developed as individuals’ ‘Build new skills’ ‘Working towards independence’
Rights (e.g., accessibility, freedom from harm/abuse and discrimination)	2%	‘Being treated with dignity, respect, equality’ ‘With no discrimination’

Most commonly, a single‐focus response related to the individuals supported being independent or living independently (coded as self‐determination). In fact, self‐determination was the most commonly coded domain overall. Least common were responses that could be coded within the rights, material well‐being and interpersonal relationships domains.

There were no significant associations at *p* < 0.05 between whether the people the staff members supported were receiving good active support or mixed/weak active support and whether quality of life was mentioned in general or as any specific domain. Staff who referred to indicators of the self‐determination domain were supporting individuals with significantly higher average adaptive behaviour scores (Mann–Whitney *Z* = −2.606, *p* = 0.009, *n* = 88). There were no other differences for any other domains.

### 3.3 How Do Staff Describe Their Role and the Support They Provide to People and Do Descriptions Differ When They are Working in a Service Providing Good Active Support?

Twenty‐six percent of responses could be coded for the approach to support that was used: 30% of these referenced normalisation (e.g., ‘the main aim is to support the service users to live a normal life to fit in the community environment’); 48% included reference to being person‐centred or using person‐centred approaches (e.g., active support or person‐centred planning); and 22% referred to the more general approach of being positive and/or encouraging.

Staff differed in how they described the way they worked to achieve the aims of the service. Table 4 presents the verb and object used for the 73 people who answered the question. General reference to ‘supporting (people)’ was most common. Fewer people referred to enabling or empowering the people they supported or to improving quality of life of people supported in some way. A small number of people used less enabling and more directive terms such as ‘to *make* people …’ or to ‘*keep* people’ (the latter usually with reference to safety).

**TABLE 4:** How respondents described the way they or the service worked—Proportion of staff using each verb (*n* = 73).

Verb used (object)	%
Support (people)	41%
Enable, empower, encourage and work with (people)	16%
Improve, build, promote, make better and develop (QOL)	16%
Provide/give (care/support)	14%
Give, offer, support, create and maintain (QOL)	11%
Help or assist (person)	9%
Allow (people)	3%
Make (people)	3%
Ensure (QOL)	3%
Meet (needs)	3%
Care	3%
Keep (people)	1%
Teach (skills)	1%
Tailor (support)	1%

There was no association at *p* < 0.05 between how staff referred to how they worked and whether they worked in teams where active support was consistently good.

### 3.4 How Do Staff Describe the Type of Skilled Support Needed by the People They Support and Do Descriptions Differ When They Are Working in a Service Providing Good Active Support?

Seventy‐three staff provided a response to this question. Forty percent reported that the people they supported needed skilled support in the area of communication (which included intensive interaction, support for choices and decision making). Fourteen percent of staff reported that skills in the management of behaviour were needed (e.g., ‘diffusing any challenging behaviour before it escalates’). Skills needed in accessing the community were reported by 7% (e.g., ‘support to access the community—no road sense’). Skills in understanding and supporting autistic people were suggested by one fifth of staff. This included some specific references to providing a low arousal approach (e.g., ‘low arousal training’) and empathy (e.g., ‘empathy’). Just over one quarter of staff (27%) mentioned skills that were required to support people in terms of their physical well‐being and health and safety (including medication, moving and handling, food hygiene, first aid hoisting and epilepsy support). Nineteen percent identified aspects related to teaching or developing the skills of the people they supported (e.g., ‘improve their lives and skills’), and 36% mentioned skills related to some other element of needs or support (e.g., cultural, religious, emotional, employment and housing)

Staff from services rated as providing weak or mixed active support were more likely to report that the people they supported needed skilled support in the area of communication (*Χ* ^2^ = 9.088, *p* = 0.003, df 1).

### 3.5 What Do Staff Report as Important in Helping Them to Become Skilled and Does This Differ When They are Working in a Service Providing Good Active Support?

All staff who answered the question on whether they felt they provide skilled support responded that they felt they were at least partially skilled. Of the 77 people who gave further explanation, 21% did not provide a detailed or clear enough answer to be coded. Fifty‐seven percent referred in very general or simple terms to the fact they had training (e.g., ‘through training’) or to the length or variety of their experience (e.g., ‘I have worked in the field of learning disabilities for over ten years and have gained considerable skills and knowledge over the years’). Only 22% of participants gave what was coded as a somewhat more reflective or detailed answer where they implied that their skills arose from putting training into practice, using approaches over time or reflecting on experience (e.g., ‘I am trained and have experience to support our tenants; we are part of a team—anything I am not sure about one of the team members will be able to help. I know our tenants and their equipment we use works and [know] how to use/work equipment’).

Of those who referred to training and experience, 50% referred to just training, 13% referred only to experience and 29% referred to both training and experience. There were no significant differences at *p* < 0.05 between staff in services providing good active support and services providing mixed or weak support in terms of whether people felt partially or fully skilled or how they explained their answer in terms of whether they mentioned training, experience or both.

In terms of whether there was any differentiation between skilled and unskilled staff in terms of the tasks they undertook, very few staff answered this question in a way that could be usefully coded, although 79% of staff reported that there *was* a differentiation at least some of the time. From those answers that could be coded, the tasks reported as needing more skilled staff were those where specific training was required: ‘unskilled staff implies “untrained” staff—such staff would not for example administer medication or move and handle via aides’. Other differentiated tasks included ‘managing customers in a critical situation’, having ‘administrative responsibilities’ and providing ‘supervision’ to other staff.

When asked about how they were helped to provide skilled support, 85% of the 81 people who answered the question again mentioned training but often in combination with other elements.

Training and development during supervision the strengths and needs of training are discussed, feedback from people we support and staff team discussion and opportunities to look at other services. I also remain close to British Institute of Learning Disabilities, MENCAP

Forty percent referred to support from others or learning from others in some way (e.g., observing experienced staff, support from professionals, being part of a team and staff communication). Ten percent mentioned having access to or reading support plans, risk assessments or other elements of paperwork. Just over one fifth of participants referred to an element that could be coded as practice leadership—for example, supervision, mentoring from manager, team meetings and discussions. However, no one mentioned elements such as coaching, being observed or being given feedback on their practice.

There were no significant associations at *p* < 0.05 between those who mentioned any element of practice leadership in terms of what helped them to be skilled (e.g., supervision, team meetings, observations and coaching) and whether they worked in a service providing good active support. There was also no significant association at *p* < 0.05 between those who said training helped them to be skilled (85% of staff) and whether they worked in a service providing good active support (50% of staff).

Table 5 presents the proportion of staff who reported receiving each type of training overall, and the proportion who said that training had helped them to provide skilled support. As can be seen, the training most commonly reported were person‐centred planning, understanding challenging behaviour (not positive behavioural support) and understanding autism. There were no significant associations at *p* < 0.05 between whether staff reported receiving any of the specific training courses and whether they reported that training helped them provide skilled support.

**TABLE 5:** Percentage of staff reporting each type of training for overall sample and for those who reported that training made them skilled.

Training	Percentage of those who said training helped them provide skilled support (*n* = 69)	Overall sample (*n* = 97)
Person‐centred planning	81%	82%
Other understanding CB	72%	71%
Understanding autism	62%	62%
Other managing CB	58%	62%
Positive behaviour support	57%	55%
Active support	55%	60%
Had classroom‐based training only	*54%*	*54%*
Had hands‐on and classroom‐based training	*39%*	*35%*
SPELL framework training	52%	51%
Risk assessment	52%	55%
Supporting autistic people	48%	49%
Person‐centred thinking	48%	50%
Risk management	39%	40%
Intensive interaction	35%	40%
Augmentative and alternative communication	33%	34%
Makaton, BSL or other sign languages	32%	34%

Training	Percentage of those who worked in a service providing good active support (*n* = 46)	Overall sample (*n* = 97)
Person‐centred planning	80%	82%
Other understanding CB	65%	71%
Understanding Autism	65%	62%
Active support/person‐centred active support	63%	60%
Had classroom‐based training only	*58%*	*54%*
Had hands on and classroom‐based	*26%*	*35%*
Other managing CB	54%	62%
Supporting autism	50%	49%
Positive behaviour support	48%	55%
Person‐centred thinking	46%	50%
Risk assessment	43%	55%
Risk management	30%	40%
Intensive interaction	28%	40%
SPELL framework training	26%	51%
Makaton, BSL or other sign language	26%	34%
Augmentative and alternative communication	17%	34%

There was a very similar picture in terms of the training reported by staff working in services providing good support (see Table 5). After Bonferroni corrections, there was just one significant association between training types and whether staff were working in a service providing good active support—in services providing good active support there were significantly fewer staff who reported having had training in augmentative and alternative communication (*Χ* ^2^ = 10.78 [1] *p* = 0.001). This is likely to be related to the fact that staff working in services providing good active support tended to be supporting people with lower support needs (*z* = 5.508, *p* < 0.001) and were less likely to report that the people they supported needed skilled support with communication (*Χ* ^2^ = 9.088 [1] *p* = 0.003). Those who reported that the people they supported needed skilled support for communication were working with people with higher support needs (*z* = 2.737, *p* = 0.006). There were no significant associations at *p* < 0.05 between whether staff reported training in positive behaviour support or other training in understanding or managing challenging behaviour and whether they had reported that the people they supported needed skilled support in this area. The same was true for those that reported that the people they supported needed skills related to understanding and supporting autistic people.

## 4 Discussion

### 4.1 Key Findings

The majority of participants reported that maintaining/improving quality of life of people they supported was a key service aim, at least in some way. The people supported were the expressed focus of services and of staff activity for the most part, and their outcomes were the priority. This is consistent with earlier research such as Windley and Chapman (2010) and Gomes and McVilly (2019). Being able to improve the quality of life and in particular support the personal development of individuals has been found to be important for staff retention (Stevens et al. 2019). However, in our study, very few respondents used the term ‘quality of life’ but talked either in more general terms such as ‘a full life’ or focused on one or two very specific elements. The elements more commonly mentioned—independence/growing in skills, experiencing choice and control, and social inclusion—are those that have been the focus of Learning Disability policy in the United Kingdom since the Valuing People White Paper (Department of Health 2001). Interestingly, the fourth principle of Valuing People, ‘Rights’, was rarely mentioned by staff. In this White Paper, the requirement for all people with intellectual disabilities to have a person‐centred plan was introduced, with further guidance 2002 (Department of Heath). In this study, person‐centred planning was the most commonly reported training from staff members.

In terms of training received, the least commonly reported training was training related to augmentative or alternative communication (AAC). Less than one third of respondents reported ever receiving training in any method of AAC, despite the fact that the study was focused on services supporting individuals with more intensive and complex support and communication needs.

Though it is reassuring that staff appeared to be able to express the aims of their service in line with policy, there was little evidence from our observational study (Beadle‐Brown et al. 2020) that such expressed service aims were being implemented in practice. Even where staff had had training in person‐centred planning and understanding behaviour that challenges and were able to express the importance of supporting good outcomes for the people they supported, those individuals were not necessarily receiving good outcomes or consistently good, person‐centred support (Beadle‐Brown et al. 2016). Similarly, support was not seen to be better in services where staff referred to any of the Quality of Life domains compared to services where they did not. Finally, the responses given by staff about the type of skilled support the people they supported needed in general did not relate to supporting/improving quality of life. Instead, they were focused more on meeting needs and the management of behaviour. Overall, very few staff provided more detailed responses or referred to any of the approaches, which are often viewed as markers of good practice.

All staff reported themselves to be at least partially skilled, and the majority explained what they meant by ‘skilled’ in terms of either having attended training in general—few referred to specific types of training—or in terms of having worked in their role for an extended period of time. The latter finding is consistent with the findings from other studies such as Windley and Chapman (2010) and Bradshaw and Goldbart (2013) where staff reported that direct knowledge of the individual and learning from watching other staff and trial and error were key ways in which they learnt to do their job. Although experience is clearly important, learning from other staff is only really helpful if the other staff are providing support in line with organisational values, policies and what is accepted as good practice. In addition, learning by ‘trial and error’ can be effective but only if the person doing the ‘trial’ is the one impacted by the ‘error’—if trial and error impacts negatively on someone else then may not be an effective or appropriate way to learn. The emphasis on training was also highlighted in the question about differentiation in the tasks that a ‘skilled’ member of staff would undertake compared to an ‘unskilled’ member of staff. Although only a small number of differences were identified, such as administering medication and moving and handling, many of the responses to this question also highlighted that having training equated to being ‘skilled’.

Although training was suggested by staff to be the key to skilled support, there was no evidence that the training individuals reported had an impact on the quality of support as observed in these services. In fact, some of the analyses appeared to indicate that if staff had training in AAC, the quality of support people received was lower. It is possible that this is an artefact of the communication needs being higher in services which were supporting people who had more complex needs. This is consistent with previous research that found that, in general, there is an inverse relationship between support needs and the quality of support, especially if there is no intervention to improve the quality of support such as training in active support and the implementation of practice leadership (Mansell and Beadle‐Brown 2012; Esteban et al. 2021). Although some respondents mentioned that team meetings and supervision helped them to provide skilled support, very few people mentioned other elements of practice leadership such as coaching. This is consistent with interviews with managers in these services who rarely referred to elements of practice leadership when discussing how they helped staff to be skilled (Bradshaw et al. 2018).

Apart from active support training, the *nature* of the training staff members received was not explored. However, there was little indication that staff had received hands‐on training, which we know is essential if support staff are to embed and implement the training they receive in the classroom or online (Jones et al. 1999; Mansell and Beadle‐Brown 2012; Flynn et al. 2018; Mullins et al. 2023).

### 4.2 Limitations

A relatively small sample of staff returned the survey, with variability in the number of surveys returned per service and in the completeness of the free text questions analysed for this paper. However, our response rate of 25% is at the upper end of the recommended response rate suggested by Wu et al. (2022) to provide fairly confident estimates with sample sizes of less than 500 respondents. Given our methodology is primarily qualitative and exploratory, our survey was paper based rather than online, and the research was conducted at a time of high vigilance and low morale in services due to recent scandals in the media, a 25% return rate with a good spread across service settings was felt to be satisfactory and worth reporting. In addition, our intention was not to recruit a representative sample of staff in services more generally but to look at the views and experiences of staff in the services where we had collected data on the quality of those services. We have data from staff across 28 services, with roughly equal numbers of both services and staff in each group (weak/mixed vs. good active support). Only seven services returned no staff questionnaires at all. We recognise that there may be between‐service differences in terms of the support and training (both type and content) provided for staff even within the same organisations and that staff may not hold the same views even if they support people with similar levels of support needs. Within organisation differences are likely to reflect, at least in part, a lack of a shared vision and strong organisational culture.

As surveys were anonymous, there was no way of checking our interpretations of responses. Although in‐depth qualitative interviews would have allowed greater exploration of staff views, managers in the same services who were interviewed also found it hard to define skilled support (Bradshaw et al. 2018). In general, the managers also did not share the ways of defining, implementing, measuring or sustaining skilled support in a manner consistent with evidence‐based practice such as person‐centred active support.

Finally, it is acknowledged that there may have been a clustering effect but, due to the small sample size, statistical techniques to eliminate the impact of clustering, such as multilevel modelling, were not possible.

### 4.3 Implications

Quality of life is generally better where individuals are supported to be actively engaged in a range of tasks, activities and interactions in different life areas, at home and in the community. Depending on the needs of the individuals supported, staff are likely to require additional skills such as augmentative and alternative communication, understanding and supporting autistic individuals, intensive interaction or positive behaviour support (Mansell and Beadle‐Brown 2012; Bradshaw 2021). The *right* support can substantially compensate for higher levels of disability and complexity of needs to produce good quality of life outcomes (Mansell and Beadle‐Brown 2012).

Although there has been no substantial change in learning disability policy in the United Kingdom since 2001, the evidence points to implementation gaps between policy and practice and between expressed values of staff and practice. This implementation gap is widely acknowledged, including in adult social care (Scottish Government 2021). There are a number of factors that potentially interact to explain the observed gap in implementation.

Staff are less likely to have training that helped them put values into action, especially in terms of supporting those with higher and/or more complex needs. Particularly noticeable in this study was the fact that only a minority of staff reported training related to communication, even though staff reported many people they supported needing skilled support in this area. Training had typically been only classroom‐based or online learning, despite the fact that both research and theory have highlighted the importance of hands‐on/in situ training for approaches to practice (e.g., Jones et al. 2001; Mansell and Beadle‐Brown 2012). Attention needs to be given to developing training that better supports staff to put into practice the values they have been taught to improve the outcomes experienced by those they support. One such approach (practice leadership) involves the use of coaching, mentoring and support to help staff to improve their performance. There has been an increased focus on Practice Leadership in the United Kingdom with the publishing of information on a Practice Leadership Role (Department of Heath and Social Care 2024).

Research has shown that practice leadership is a key factor in determining the quality of support provided and thus the outcomes experienced by those receiving services. Putting this into practice may be difficult. First, austerity resulted in fewer front‐line managers and therefore more multisite management, meaning that managers were less present and therefore unable to provide practice leadership in these services (Bradshaw et al. 2018). We know from other research that even where practice leadership is adopted, barriers exist including a lack of stability within staff teams (Deveau and Rickard 2024). Second, cuts to services following austerity reduced the funding available and resulted in managers managing many more services than prior to austerity; COVID‐19 further contributed to difficulties in being able to recruit and train staff due to shortages in social care staff (Hatton et al. 2023).

## Ethics Statement

Ethical approval was gained from the Social Care Research Ethics Committee.

## Conflicts of Interest

The authors declare no conflicts of interest.

## Data Availability

The data that support the findings of this study are available from the corresponding author upon reasonable request.
